# A 12-Year Comparative Analysis of Hodgkin and Non-Hodgkin Lymphomas in Lebanon: Trend Characteristics and 10-Year Projections

**DOI:** 10.7759/cureus.8412

**Published:** 2020-06-02

**Authors:** Ali H Abdel Sater, Mohammad Jalloul, Marwan Zein, Zeina Lakis, Hussein H Khachfe

**Affiliations:** 1 Medicine, Baabda Governmental Hospital, Beirut, LBN; 2 Medicine, Faculty of Medical Sciences, Lebanese University, Beirut, LBN; 3 Medicine, Faculty of Medical Sciences, Lebanese University, Beirut , LBN; 4 General Surgery, American University of Beirut Medical Center, Beirut, LBN

**Keywords:** hodgkin lymphoma, cancer, lebanon, non-hodgkin's lymphoma

## Abstract

Background

Hodgkin lymphoma (HL) and non-Hodgkin lymphoma (NHL) are two common malignancies worldwide and in Lebanon. Analysis of their trends plays a crucial role to better understand their origins and risk factors. This study will probe incidences of both types of lymphomas from 2005 to 2016, aiming to compare between the two malignancies according to age and sex and plot projections until 2026.

Methods

HL and NHL cases from 2005 to 2016 were collected from the National Cancer Registry of Lebanon. Data was stratified according to age and sex. Age-specific and age-standardized incidence rates were analysed using joinpoint regression; 10-year projections were predicted based on logarithmic models.

Results

Between 2005 and 2016, NHL was significantly more common than HL. NHL was higher in both genders. HL showed a bimodal age distribution while NHL peaked in elderly patients. NHL incidence rates in males increased significantly from 2005 to 2014 while HL incidence rates showed an insignificant rise. Over the next 10 years, NHL and HL cases are expected to increase in Lebanon.

Conclusion

HL and NHL are on the rise in Lebanon. Extensive research into the main factors contributing to these lymphomas is crucial in the fight against them. More efforts must be done by the government and health organizations to better control the disease.

## Introduction

Cancer constitutes the second most common cause of death globally, coming second only to cardiovascular related diseases [1]. In 2018, lymphoid neoplasms were responsible for 3.2% of all new cancers worldwide [2]. Lymphomas are a group of malignant tumors that originate from aberrant proliferation of lymphoid cells and their precursors [3]. They are broadly classified by the World Health Organization (WHO) into several groups based on cell of origin of the tumor, morphology, immunophenotype, and genetic findings [4]. Lymphoid tumors do not only include Hodgkin lymphomas (HLs) and non-Hodgkin lymphomas (NHLs), but also plasma cell neoplasms and lymphoid leukemias [5].

Risk factors associated with lymphomas include age, gender, ethnicity, and geographic location [6]. Other risk factors include chemicals and radiation exposure, immune system deficiency, HIV infection and family history [7].

Lebanon is a developing country in the Middle East and North African (MENA) region with a population of around six million people as of 2018 [8]. The Ministry of Public Health (MoPH) of Lebanon has established a database that collects new cases of cancers from hospital registries, physician reports, and public and private laboratories. It is known as the National Cancer Registry (NCR) of Lebanon [9].

Using data from 2005 to 2016 collected by the NCR, this study aims to explore the epidemiological characteristics of both HLs and NHLs and compare them among different age groups and genders. The study will also discuss the possible risk factors of HL and NHL in Lebanon, and plot projections of incidence rates of both malignancies until 2026.

## Materials and methods

Data and records concerning the different types of lymphomas were extracted from the Lebanese NCR database provided by the Lebanese MoPH [9]. This was done using the International Classification of Diseases, 10th Revision (ICD-10) for HL (C81) and NHL (C82-C85 and C96).

The NCR was screened for the time period 2005-2016, from which age-specific and age-standardized incidence rates, expressed per 100,000 population, were computed. The age-specific incidence rate represented the number of new cancer cases that occurred during a specific time period in a population of a specific age and sex group divided by the number of midyear population of that age and sex group. The age-standardized rates to the world population, henceforth ASR(w)s, were the incidence rates that would have been observed in our studied population if they have had the same age composition as a reference population.

The calculated age-specific rates (ASRs) and ASR(w)s were analysed using joinpoint regression analysis with a significance level of 0.05. The joinpoint model provides detailed information of the trends of the studied hematological malignancies, including the annual percent change (APC) of each type of the selected cancers, over the specified time period.

Using two-tailed t-tests, we also compared the means between different groups of age and genders to search for any significant difference in the incidence rates, considering a P-value < 0.05 as significant.

By exploiting the extracted and analysed data listed above and using a logarithmic model, we estimated the predictions of HL and NHL incidence rates in Lebanon until 2026. We found the logarithmic model to be the most biologically credible model to follow the trends of cancer over time [10]. The criteria for characterizing the observed trends followed the National Cancer Institute (NCI) guidelines stating that if the APC > 0.5% the trend is said to be rising; if the APC < −0.5%, the trend is judged to be declining; and if the −0.5% ≤ APC ≤ 0.5%, the trend is judged to be stable [11].

Regarding the need for an Institutional Review Board (IRB) approval, our study is a descriptive epidemiological study with openly accessible, deidentified data available online and published by the Lebanese MoPH. Therefore, no IRB approval was required.

## Results

Over the studied period from 2005 to 2016, a total of 126,480 new cancer cases were reported by the NCR. NHL was found to be the seventh most common cancer in Lebanon contributing to 5.5% of the total malignancies registered, while HL constituted 1.7% of new cancer cases during the specified time. An average of 578 NHL cases and 176 HL cases were indexed every year.

Males were significantly more affected by HL (P-value = 0.01, 95% CI) and NHL (P-value < 0.01, 95% CI). NHL was significantly more prevalent than HL in both genders (P-value < 0.01, 95% CI). HL ASR(w)s of males averaged 4.2 cases per 100,000 and that of females centered at 3.3 cases per 100,000, as shown in Tables 1, 2, while NHL ASR(w)s observed had a mean of 14.8 and 12.0 in males and females, respectively (Tables 3, 4). In males, incidence rates recorded peaks of 5.5 HL cases per 100,000 in 2016 and 16.9 NHL cases per 100,000 in 2014, whereas in females, rates mounted to their highest values in 2014 with 3.9 HL cases per 100,000 and 14.3 NHL cases per 100,000.

**Table 1:** Trend analysis for Hodgkin lymphoma ASR per 100,000 in males by age group per year in Lebanon from 2005 to 2016 ASR, age-specific rate; APC, annual percent change *APC significantly different than zero.

Year	Average ASR	ASR															
		0-4y	5-9y	10-14y	15-19y	20-24y	25-29y	30-34y	35-39y	40-44y	45-49y	50-54y	55-59y	60-64y	65-69y	70-74y	75+y
2005	3.5	0.6	0	3.3	5.4	5.8	3.9	3.5	5.6	3.3	2	3.4	4.3	3.1	5	8.3	8.3
2006	3.9	0	0.5	3.2	6.7	4.3	5.8	2.7	4	4.1	5	8.9	5.6	0	1.6	10.2	8.2
2007	4.3	0	1.2	3.1	4.8	4.4	4.5	9.9	3.3	8.3	4	4.6	7	7.4	7.9	1.8	1.4
2008	4.1	0	1.4	1.8	6.2	4	2.4	1.9	4.9	6.8	10.7	7.6	5.5	2.9	8.9	10.2	0
2009	3.9	0.6	1.9	1.8	0.9	5.4	8.8	1.9	4.8	2.9	3.5	3.8	9.5	7.2	6.5	0	14.9
2010	5.1	0	3.2	1.3	8.8	5.4	9.8	5.6	4.7	3.8	4.6	11.1	5.3	2.8	6.4	7.4	16.5
2011	3.2	0.5	1.3	3	1.4	3.8	2.8	4.3	1.5	3.7	7.9	2.4	6.6	5.6	10.5	2.4	3.6
2012	4.8	0	0.9	1.7	5.8	9.4	5.6	3.6	6.1	9.1	12.2	5.9	3.9	6.9	2.1	0	5.3
2013	3.6	0.4	1.5	1.5	3.9	3.9	4.9	4.7	4.6	5.6	5	3.3	6	2.6	7.8	6.8	1.7
2014	5.1	0.3	0.6	1	2.5	4	2.2	7.4	3.5	5.9	12.7	5.1	14.6	7.4	20.6	12.9	19.3
2015	3.8	0	1.2	0.9	6.1	7.4	7.1	3.5	3.4	2.8	7	5.9	4.4	3.6	0	8.5	4.7
2016	5.5	0	2.4	1.2	4.6	5.2	7.4	5.2	6.4	9.4	5.4	12	14.5	3.7	9.3	8.5	15.9
APC	1.9	-	-	-10.2*	-2	1.5	1.7	3.9	-0.1	2.9	8.2	2.3	5.1	-	-	-	-
P-value	0.2	-	-	<0.05	0.7	0.6	0.7	0.4	1	0.5	0.1	0.6	0.2	-	-	-	-
CI	[-1.1, 5.1]	-	-	[-14.6, -5.5]	[-14.1, 11.9]	[-3.8, 7.1]	[-7.6, 12.0]	[-5.4, 14.0]	[-7.4, 7.8]	[-5.4, 11.9]	[-1.3, 18.6]	[-7.2, 12.8]	[-2.8, 13.7]	-	-	-	-

**Table 2:** Trend analysis for Hodgkin lymphoma ASR per 100,000 in females by age group per year in Lebanon from 2005 to 2016 ASR, age-specific rate; APC, annual percent change

Year	Average ASR	ASR															
		0-4y	5-9y	10-14y	15-19y	20-24y	25-29y	30-34y	35-39y	40-44y	45-49y	50-54y	55-59y	60-64y	65-69y	70-74y	75+y
2005	3	0	0	3.5	4.1	6.2	2.4	3.4	2.7	2	1.8	5.7	2.6	1.4	3.3	6.8	14.5
2006	2.5	0	0	1	2	5.1	6.6	3.3	0	1.3	3.6	3.3	1.3	5.4	6.5	2.2	4.1
2007	3.8	0	0	3.3	3.9	4.3	5.4	8.6	4.1	5	0.8	2	11.3	5.1	3	10.9	6
2008	2.9	0	0	2.4	5.6	4.7	4.4	3.4	2.1	1.8	6.7	5	2.6	2.9	2	4.9	0
2009	3.2	0	0.5	2.8	5	5.6	5.9	6.1	3.5	2.6	1.1	3.7	5.1	2.8	3.9	2.4	2
2010	3.6	0	0	1.9	3.9	7	5.3	6.6	1.4	3.4	9.7	4.8	1.3	8.3	1.9	2.4	5.8
2011	3.6	1.7	0.5	0.5	5.3	8.4	4.7	3.2	4.1	4.2	2.1	4.7	0	6.8	3.8	9.3	7.7
2012	2.7	0	1.9	1.8	3.8	4.4	3.1	2.1	4	3.3	2.1	0	2.4	8.1	3.7	0	5.7
2013	3.4	0.4	0	1.2	5	6.8	4	5.1	4.8	4.4	2.8	4.2	3.4	0	7.1	4.3	7.1
2014	3.9	0.4	0.3	1.7	5.9	3	5	6.2	1.1	4.7	3.4	3.9	7.3	4.7	11.7	10.2	15.2
2015	3.3	0	1.5	2.3	3.9	7.6	8.2	6	2.6	2	2.5	1.9	6.1	2.3	1.6	0	3.3
2016	3.5	0	0	1.3	4.4	5.3	6.7	5.7	2.6	3.3	3.4	2.9	6.2	9.4	3.3	4	6.7
APC	1.6	-	-	-4.8	3.1	0.2	3.6	2.4	-	5.1	3.5	-	-	-	1.3	-	-
P-value	0.2	-	-	0.3	0.2	0.9	0.2	0.5	-	0.2	0.6	-	-	-	0.8	-	-
CI	[-0.8, 4.2]	-	-	[-14.1, 5.5]	[-2.1, 8.5]	[-5.3, 6.0]	[-2.6, 10.2]	[-5.3, 10.8]	-	[-2.6, 13.5]	[-9.3, 18.2]	-	-	-	[-9.4, 13.4]	-	-

**Table 3:** Trend analysis for non-Hodgkin lymphoma ASR per 100,000 in males by age group per year in Lebanon from 2005 to 2016 ASR, age-specific rate; APC, annual percent change

Year	Average ASR	ASR															
		0-4y	5-9y	10-14y	15-19y	20-24y	25-29y	30-34y	35-39y	40-44y	45-49y	50-54y	55-59y	60-64y	65-69y	70-74y	75+y
2005	3	0	0	3.5	4.1	6.2	2.4	3.4	2.7	2	1.8	5.7	2.6	1.4	3.3	6.8	14.5
2006	2.5	0	0	1	2	5.1	6.6	3.3	0	1.3	3.6	3.3	1.3	5.4	6.5	2.2	4.1
2007	3.8	0	0	3.3	3.9	4.3	5.4	8.6	4.1	5	0.8	2	11.3	5.1	3	10.9	6
2008	2.9	0	0	2.4	5.6	4.7	4.4	3.4	2.1	1.8	6.7	5	2.6	2.9	2	4.9	0
2009	3.2	0	0.5	2.8	5	5.6	5.9	6.1	3.5	2.6	1.1	3.7	5.1	2.8	3.9	2.4	2
2010	3.6	0	0	1.9	3.9	7	5.3	6.6	1.4	3.4	9.7	4.8	1.3	8.3	1.9	2.4	5.8
2011	3.6	1.7	0.5	0.5	5.3	8.4	4.7	3.2	4.1	4.2	2.1	4.7	0	6.8	3.8	9.3	7.7
2012	2.7	0	1.9	1.8	3.8	4.4	3.1	2.1	4	3.3	2.1	0	2.4	8.1	3.7	0	5.7
2013	3.4	0.4	0	1.2	5	6.8	4	5.1	4.8	4.4	2.8	4.2	3.4	0	7.1	4.3	7.1
2014	3.9	0.4	0.3	1.7	5.9	3	5	6.2	1.1	4.7	3.4	3.9	7.3	4.7	11.7	10.2	15.2
2015	3.3	0	1.5	2.3	3.9	7.6	8.2	6	2.6	2	2.5	1.9	6.1	2.3	1.6	0	3.3
2016	3.5	0	0	1.3	4.4	5.3	6.7	5.7	2.6	3.3	3.4	2.9	6.2	9.4	3.3	4	6.7
APC	1.6	-	-	-4.8	3.1	0.2	3.6	2.4	-	5.1	3.5	-	-	-	1.3	-	-
P-value	0.2	-	-	0.3	0.2	0.9	0.2	0.5	-	0.2	0.6	-	-	-	0.8	-	-
CI	[-0.8, 4.2]	-	-	[-14.1, 5.5]	[-2.1, 8.5]	[-5.3, 6.0]	[-2.6, 10.2]	[-5.3, 10.8]	-	[-2.6, 13.5]	[-9.3, 18.2]	-	-	-	[-9.4, 13.4]	-	-

**Table 4:** Trend analysis for non-Hodgkin lymphoma ASR per 100,000 in females by age group per year in Lebanon from 2005 to 2016 ASR, age-specific rate; APC, annual percent change *APC significantly different than zero.

Year	Average ASR	ASR															
		0-4y	5-9y	10-14y	15-19y	20-24y	25-29y	30-34y	35-39y	40-44y	45-49y	50-54y	55-59y	60-64y	65-69y	70-74y	75+y
2005	11.3	0	1.2	1	1.6	2.1	6.1	2.7	9.4	14.1	14.7	22.6	26.2	31.8	43.1	81.4	72.5
2006	11.9	1.3	2.3	2.4	3.6	0	7.2	3.3	4.6	11.3	13.6	25.6	37.4	29.9	45.7	64.6	85.7
2007	10.2	0	1.8	0	2.2	2.1	4.8	6.6	6.8	12.2	13.5	27.3	32.8	27.8	35.7	52.7	51.1
2008	11.6	0	1	0.5	1.5	4.2	8.8	5.7	5.7	9.6	14.5	21.1	22.1	35.9	50	63.5	103
2009	12	0.6	1	1.9	2.5	4.1	2.7	6.7	11.9	6.9	19.7	30.6	24.3	28.3	45.2	76.8	81.4
2010	12.4	0.6	1.9	0.9	2.4	4.5	5.3	4.9	9	14.4	24.7	16.8	27.6	36.1	38.6	58.9	107.2
2011	12.8	0.6	1.9	1.8	1.9	4.9	5.7	9.1	6.8	13.3	20.1	31.9	38.3	34.1	36.1	53.3	86.3
2012	13.7	1.1	0.5	0.9	1.9	3.4	10.7	8.5	2.7	9.8	31.2	30.2	41.3	36.2	52.3	43.3	96.1
2013	10.8	0.9	1.2	1.5	3.3	3	3.5	4.2	8.3	6.6	14	22.3	33.7	21.4	42.3	53.8	96.1
2014	14.3	0.7	0.6	0.3	2.9	3	3.1	6.2	11.7	8	27.7	30.8	41.6	41.3	65.3	47	120
2015	12.5	0	0.9	1	1.1	3.2	3.7	5.2	4.7	9.8	27.1	23.7	27.6	41.7	65.7	54.1	94.5
2016	11	1.1	0	1	1.8	1.9	3.5	4.1	6.3	6.6	22.8	28.9	26	43.5	46.4	42.5	63.6
APC	0.9	-	-	-	-1.6	-	-5.3	2.9	-1.1	-4.6*	6.1*	1.3	1.0	2.6	2.8	-4.2*	1.9
P-value	0.3	-	-	-	0.6	-	0.1	0.4	0.8	<0.05	<0.05	0.4	0.6	0.1	0.1	<0.05	0.4
CI	[-0.9, 2.7]	-	-	-	[-7.9, 5.2]	-	[-12.1, 2.1]	[-3.9, 10.1]	[-9.0, 7.4]	[-8.9, -0.1]	[1.5, 11.0]	[-2.4, 5.1]	[-3.1, 5.3]	[-1.0, 6.3]	[-0.6, 6.3]	[-6.7, -1.6]	[-2.5, 6.5]

HL demonstrated a bimodal distribution with two peaks, the first being between the ages of 20 and 40 and the second being in people older than 60, while 76% of NHL patients were 40 years or older. The group that had the most number of cases in both cancer types consisted of males older than 75 years with an average incidence of 8.31 HL cases per 100,000 and 92.8 NHL cases per 100,000. The group with the lowest incidence in both malignancies was the one aged 0-4 years with average rates of 0.2 HL cases per 100,000 in males and 0.57 NHL cases in females.

**Figure 1:**
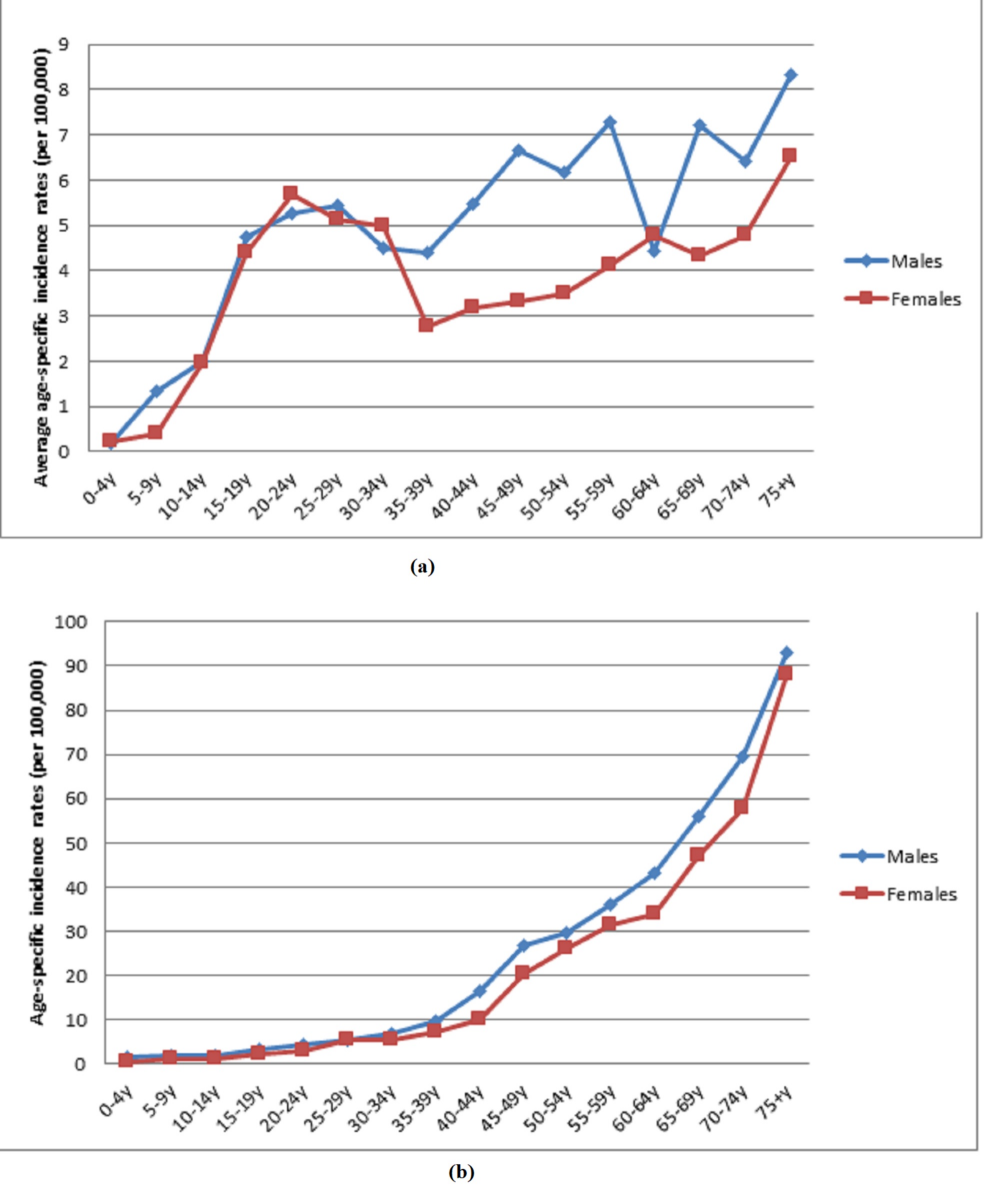
Gender and average age-specific incidence rates (per 100,000 population) for (a) Hodgkin and (b) non-Hodgkin lymphoma in Lebanon from 2005 to 2016

**Figure 2:**
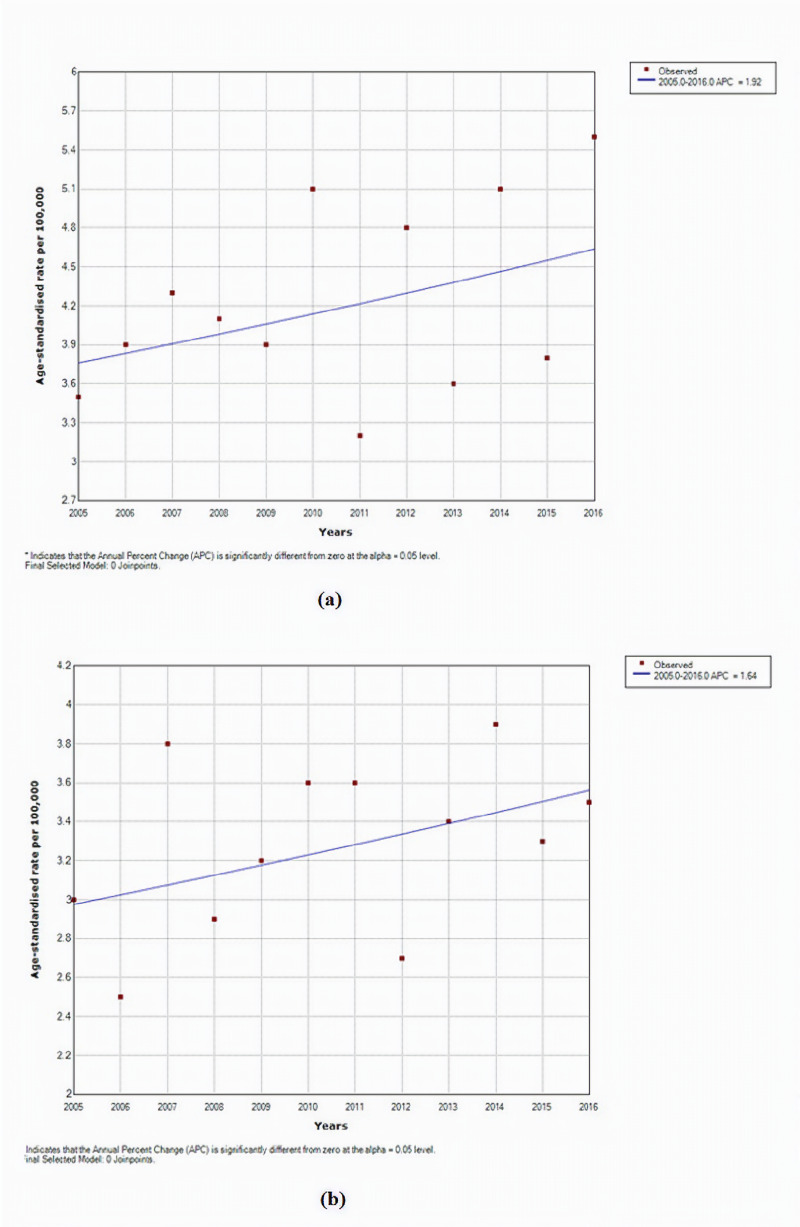
Trends of age-specific rates for Hodgkin lymphoma among (a) males and (b) females in Lebanon from 2005 to 2016

**Figure 3:**
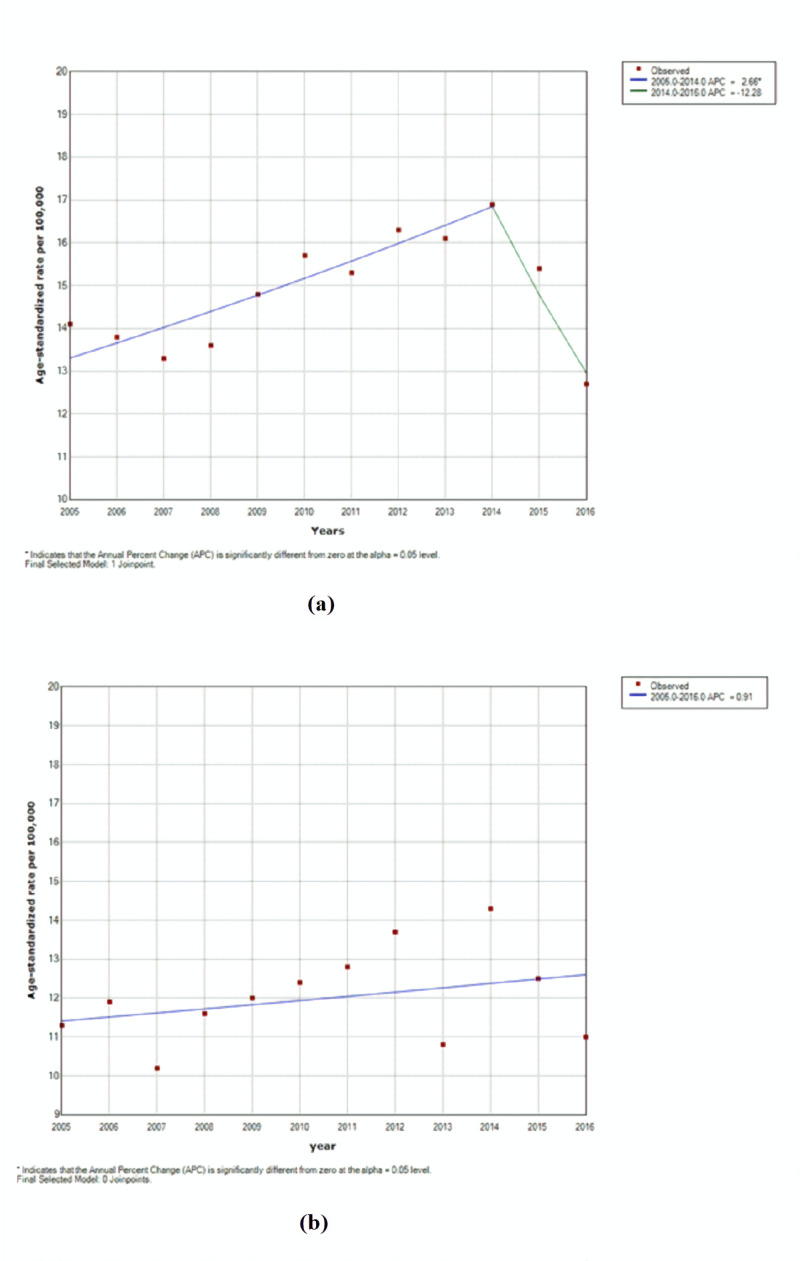
Trends of age-specific rates for non-Hodgkin lymphoma among (a) males and (b) females in Lebanon from 2005 to 2016

Throughout our study, there was no significant change in HL incidence rates in both males (P = 0.2, CI [-1.1, 5.1]) and females (P = 0.2, CI [-0.8, 4.2]) as seen in Figures 1, 2. However, a significant increase was noted in NHL rates in males from 2005 to 2014 (P < 0.05, CI [1.4, 3.9]) (Figures 2, 3). As for age-specific incidence rates, studying both genders revealed a significant decrease in HL cases in males aged 10 to 14 (P < 0.05, CI [-14.6, -5.5]) (Tables 1, 2). In females, age-specific trends of NHL showed a significant decrease in rates for age groups 40-44 years (P < 0.05, CI [-8.9, -0.1]) and 70-74 years (P < 0.05, CI [-6.7, -1.6]) and a significant increase for age group 45-49 years (P < 0.05, CI [1.5, 11.0]) throughout the 12 years (Tables 3, 4).

Relying on our 10-year predictions, NHL incidence rates are expected to significantly rise from 2016 to 2026 with an APC of 1.85 in males and 1.49 in females (P < 0.05, CI 95%) (Table 5). Moreover, no significant variation is expected in HL rates for both males and females till 2026 (Table 6).

**Table 5:** Ten-year prediction of incidence per 100,000 of non-Hodgkin lymphoma in both males and females and their APC APC, annual percent change *Significantly different than zero.

Sex	2016 (year)	2021 (year)	APC	Trend	2026 (year)	APC	Trend
Male	12.7	16.4	3.98	Rising	17.1	1.85*	Rising*
Female	11	13.1	2.76	Rising	13.7	1.49*	Rising*

**Table 6:** Ten-year prediction of incidence per 100,000 of Hodgkin lymphoma in both males and females and their APC APC, annual percent change

Sex	2016 (year)	2021 (year)	APC	Trend	2026 (year)	APC	Trend
Male	5.5	4.3	-1.46	Declining	6.1	1.39	Rising
Female	3.5	3.3	0.04	Stable	3.8	1.19	Rising

## Discussion

Of the 9,048 new cases of lymphoma reported in Lebanon from 2005 to 2016, HL accounted for 24% and NHL for 76%. These results are consistent with the epidemiological profiling of these malignancies done previously in Lebanon [12]. NHL was the seventh most common malignancy during the time period studied, which is also consistent with previous reports from the country [10].

Similar to previous research, lymphoid malignancies affected males significantly more than females in our study [13,14]. However, female predominance might be noted in some subtypes of NHL, like marginal zone and follicular lymphoma [15]. NHL was significantly more prevalent in both genders [14]. Sex predilection in lymphomas may stem from the fact that environmental exposures and working conditions that are often linked to uncontrolled lymphoproliferation tend to occur in male-driven careers such as industrial or engineering work [15]. Given that such patterns of male predominant careers are also prevalent in Lebanon, it comes as no surprise that our results mirror these previous findings. Also, it has been suggested that gravidity plays an important protective role against NHL, explaining why males were more affected by this disease [14].

Previous reports from Lebanon have shown that the age distribution of HL in both genders was found to be bimodal [12]. Lebanon witnessed improvement in the efficiency of its healthcare system from 2000 to 2015 according to a study done by Ibrahim and Daneshvar [16]. This suggests that HL’s bimodal distribution in Lebanon can be explained by the advancement of medical diagnostic studies. The implementation of highly sensitive techniques helps differentiate the various types of lymphomas and aids in diagnosing HL earlier [17]. NHL is usually more common in people older than 40 years, with the highest incidence found in people older than 70 years [18]. Therefore, the increase in life expectancy among the Lebanese population from a median age of 60.5 years in 1950 to 78.8 years in 2015 must have markedly impacted the total number of NHL cases in our study [19].

Lebanon has been witnessing an accelerating rate of obesity, increasing from 54.4% to 65.0% over the past decade, as provided by national surveys conducted by the MoPH and WHO [20]. An increase in the body mass index in most Lebanese age groups has been also described by different studies in the past decade [21]. Accordingly, this escalating rate of obesity might explain the increasing incidence of HL cases in Lebanon, given that a causative relationship has been already established between obesity and this lymphoma subtype [22].

HIV has been emerging tremendously in the MENA region during the past two decades. As reported by the Joint United Nations Programme on HIV/AIDS (UNAIDS), new HIV infections increased by 31% from 2001 to 2013, representing the highest rate among all regions in the world [23]. Some studies in Lebanon have also demonstrated a rise in the prevalence rate of HIV infections locally, in the past few years [24]. Because HIV has been highly associated with a higher risk of both HL and NHL when compared to previously healthy individuals, this increasing trend in HIV infections among the Lebanese population can help explain the increase in amounts of cases of both cancers in the country over the studied period [25].

Concerning other infectious and communicable diseases, Epstein-Barr virus (EBV) infection is a well-known risk factor for both NHL and HL [26]. Despite its wide prevalence especially in developing countries, no data is available in Lebanon. *Helicobacter pylori*, another common pathogen in developing countries, is a known risk factor for gastric mucosa-associated lymphoid tissue (MALT) lymphoma, a subtype of NHL [27]. EBV and *H. pylori* remain two well-known risk factors for lymphoma, but no sufficient local data is present to correlate them with the increasing prevalence of lymphomas in Lebanon. Therefore, more research must be published on these two pathogens to help better understand their associated diseases such as HL and NHL.

First-degree relatives of patients with lymphoma have a significantly elevated risk of developing the same malignancy, more commonly of the same subtype [28]. In addition, these correlations do not seem to be confounded by other risk factors, all of which raises the suspicion of genetic risk factors and familial predisposition to lymphomas. Furthermore, genome-wide association studies have successfully identified multiple single nucleotide polymorphisms from 41 loci predominately associated with specific subtypes [29]. More specifically, genes associated with risk for HL were most commonly located at different major histocompatibility complex (MHC)/human leukocyte antigen (HLA) loci. However, these genetic associations that are believed to increase the risk of HL are more likely multiple common risk alleles, rather than a single isolated gene [30]. In Lebanon, no studies have been conducted to complement the present knowledge of genetic risk factors for HL and NHL.

Both HL and NHL are projected to increase by 2026. This can be explained by looking back at the risk factors involved in pathogenesis of both diseases. Preventable risk factors such as environmental exposure to carcinogens are not set to decrease, and with the increase in toxic waste present in the country, one can only expect that exposure to said compounds will increase. Obesity and infections are also set to follow the other worldwide trends in increasing. These factors can help explain why the HL and NHL will continue to increase in Lebanon in the following 10 years.

To the best of our knowledge, this is the first study exploring the trends, characteristics, and projections of HL and NHL in Lebanon. Nevertheless, a few limitations are present. First, as our data was extracted from the NCR provided by the MoPH, the validity of our results depends on the reliability of the source. Second, considering that the NCR does not provide data on mortality rates or cancer staging, we were only able to identify the incidence rates of lymphoid neoplasms and evaluate their changes throughout the years.

## Conclusions

An increase in lymphoma incidence rates has been noted in Lebanon during 2005-2016. This increase can be attributed to an aging population and early cancer detection, along with other preventable risk factors. A deeper understanding of lymphoma incidence in Lebanon is largely dependent on further studies targeting the genesis of different subtypes and various modifiable and unmodifiable risk factors prevalent in the society. Ten-year projections plotted by this study denote a further increase in lymphoma cases. In front of these alarming findings, the MoPH and concerned non-governmental organizations should promote campaigns to increase society’s knowledge of different risk factors associated with lymphoid malignancies and implement measures to decrease their prevalence.
